# MicroRNA Profiling in Patients with Upper Tract Urothelial Carcinoma Associated with Balkan Endemic Nephropathy

**DOI:** 10.1155/2016/7450461

**Published:** 2016-04-27

**Authors:** Katerina Popovska-Jankovic, Predrag Noveski, Ljubinka Jankovic-Velickovic, Slavica Stojnev, Rade Cukuranovic, Vladisav Stefanovic, Draga Toncheva, Rada Staneva, Momir Polenakovic, Dijana Plaseska-Karanfilska

**Affiliations:** ^1^Research Centre for Genetic Engineering and Biotechnology “Georgi D. Efremov”, Macedonian Academy of Sciences and Arts, 1000 Skopje, Macedonia; ^2^Institute of Pathology, Faculty of Medicine, University of Nis, 18000 Nis, Serbia; ^3^Clinic of Nephrology, Medical Faculty, University of Nis, 18000 Nis, Serbia; ^4^Department of Clinical Research, Faculty of Medicine, University of Nis, 18000 Nis, Serbia; ^5^Department of Medical Genetics, Medical University of Sofia, 1431 Sofia, Bulgaria

## Abstract

Balkan endemic nephropathy (BEN) is a disease that affects people that live in the alluvial plains along the tributaries of the Danube River in the Balkan region. BEN is a chronic tubulointerstitial disease with a slow progression to terminal renal failure and has strong association with upper tract urothelial carcinoma (UTUC). There are several hypotheses about the etiology of BEN, but only the toxic effect of aristolochic acid has been confirmed as a risk factor in the occurrence of the disease. Aberrantly expressed miRNAs have been shown to be associated with many types of cancers. A number of studies have investigated the expression of microRNAs in urothelial carcinoma, mainly on urothelial bladder cancer, and only a few have included patients with UTUC. Here we present the first study of microRNA profiling in UTUC tissues from patients with BEN (BEN-UTUC) and patients with UTUC from nonendemic Balkan regions (non-BEN-UTUC) in comparison to normal kidney tissues. We found 10 miRNAs that were differentially expressed in patients with BEN-UTUC and 15 miRNAs in patients with non-BEN-UTUC. miRNA signature determined in BEN-UTUC patients differs from the non-BEN-UTUC patients; only miR-205-5p was mutual in both groups.

## 1. Introduction

Balkan endemic nephropathy (BEN) is a disease that affects people that live in the alluvial plains along the tributaries of the Danube River such as Serbia, Bulgaria, Romania, Bosnia and Herzegovina, and Croatia. BEN is a chronic tubulointerstitial disease with a slow progression to terminal renal failure and has strong association with upper tract urothelial carcinoma (UTUC) [1–6]. In areas where BEN is endemic, the incidence of UTUC is significantly higher, even 100 times, than in nonendemic regions [3–7].

There are several hypotheses about the etiology of BEN: environmental factors (nephrotoxic agents that include lead intoxication, metal and metalloids, chronic intoxication with* Aristolochia clematitis*, ochratoxin A and Pliocene lignite, and viruses), genetic predisposition (genetic polymorphisms and immunological changes), and epigenetic mechanisms (DNA methylation and specific histone acetylation). Based on previously published studies it was suggested that multifactorial aetiology is the best match with regard to BEN pathophysiology, placing the genetic predisposition of BEN [8].

The toxic effect of aristolochic acid, a nephrotoxic and carcinogenic plant alkaloid derived from* Aristolochia clematitis*, has already been confirmed as a factor in the occurrence of the disease [5, 9].

The clinical expression and pathological lesions observed at different stages of Chinese herbs nephropathy are found to be similar to the BEN except for the rate of their progression toward end-stage renal failure [10]. In 1993 Vanherweghem et al. [11] reported an unusual observation that many young Belgian women that took slimming pills containing Chinese herb subsequently developed renal failure and UTUC [11, 12]. Based on pathological characteristics, overwhelming UTUC, and identification of aristolochic acid in Chinese herbs Vanhaelen et al. hypothesized that ingestion of the* Aristolochia* species herbs may be the culprit for the epidemic in Belgium [13]. Additionally, similar studies were reported in other countries [14–17].

External environmental factors can have an influence on the genes without changing the DNA sequence itself, a mechanism known as epigenetics. The main epigenetic processes are DNA methylation, histone modifications, and miRNA interference. Epigenetic changes are required for normal development and health; they can also be responsible for some disease states. Disrupting any of the three systems that contribute to epigenetic alterations can cause abnormal activation or silencing of genes [18]. There are a few studies that involved analysis of the epigenetic factors in BEN [19, 20]. Here we focus on microRNA profiling in patients with BEN-UTUC.

MicroRNAs (miRNAs) are endogenous, noncoding RNA molecules of about 22 nucleotides in length which regulate gene expression [21]. They join the RNA-induced silencing complex to regulate their targeted messenger RNA (mRNA) by repressing mRNA translation and/or directing mRNA cleavage [22]. miRNAs play important roles in normal development, cell growth, differentiation, and apoptosis in mammals [23]. More than half of the miRNA genes are located in cancer-associated genomic regions or in fragile sites [24]. Aberrantly expressed miRNAs have been shown to be associated with many types of cancers, functioning as regulatory molecules, acting as oncogenes or tumor suppressors [25–27]. Different cancer types, stages, or differentiation states have unique miRNA expression profiles, suggesting that miRNAs can function as novel biomarkers for cancer diagnosis [28, 29]. Currently almost two thousand unique human mature microRNAs are known [30].

A number of studies have investigated the expression of microRNAs in urothelial carcinoma. The majority have been performed on the most common urothelial bladder cancer and only a few have included patients with UTUC.

Several previous investigations used miRNA microarrays to profile the miRNA expression in bladder cancer, but their results were not consistent [27, 31–33]. miRNA expression patterns have been linked to clinical outcomes in urothelial carcinoma [32, 34]. Therefore, it was suggested that single miRNA biomarkers or biomarker signatures of multiple miRNAs may improve risk stratification of patients and may supplement the histological diagnosis of urological tumors including bladder cancer [32, 35–37].

Here we present our initial results of microRNA profiling in patients with UTUC associated with BEN, as well as in patients with UTUC originating from nonendemic regions. This is the first study presenting microRNA profiles of UTUC from patients living in BEN regions.

## 2. Materials and Methods

We have performed microRNA profiling in UTUC tissues from patients living in BEN regions (BEN-UTUC), patients from nonendemic regions (non-BEN-UTUC), and normal kidney tissues (originated from patients with kidney or ureter calculosis, hydronephrosis, not caused by tumor, and accidental damage of the kidney during some injury). BEN-UTUC patients lived in settlements along the South Morava River basin known as BEN endemic region, while non-BEN-UTUC were from nonendemic rural and city regions. The study was conducted according to the Declaration of Helsinki and was approved by the Ethical Committee of the Macedonian Academy of Sciences and Arts.

Formalin fixed paraffin embedded tissues (FFPE) from patients with BEN-UTUC, non-BEN-UTUC, and normal kidney tissue were provided by the Institute of Pathology, Faculty of Medicine, University of Nis, Serbia. The histological sections stained with haematoxylin and eosin (H&E) and H&E-stained slides were used to assess histologic grade (low and high grade) [38], pathologic stage (low and high stage) [39], and growth of tumor (papillary/solid). Histopathological data were available from 12 patients (Table 1). The methodology used included extraction of total RNA, reverse transcription and microRNA real-time PCR assay, and microRNA microarray analysis.

Total RNA extraction was performed using FFPE DNA/RNA Kit (Qiagen, Hilden, Germany) following the manufacturer protocol. Quality and quantity of total RNAs were determined on NanoVue spectrophotometer (Little Chalfont, GE Healthcare, UK). RNA samples were dissolved in RNase-free water and stored at −80°C. RNA was extracted from a total of 20 samples: 7 samples were BEN-UTUC, 5 samples were non-BEN-UTUC, and 8 samples were normal kidney tissues.

Prior to the microarray analysis we checked the RNA quality of the samples by stem-loop RT followed by TaqMan PCR analysis [40] using TaqMan® MicroRNA Reverse Transcription Kit, TaqMan Universal PCR Master Mix, and RNU 44 TaqMan MicroRNA Assay (Life Technologies, Carlsbad, CA, USA). RT mix was made on ice in 10 *μ*L final volume, following manufacturer protocol. Thermal cycling conditions were 16°C for 30 minutes; 42°C for 30 minutes; 85°C for 5 minutes. Real-time PCR assay was performed in duplicate in a total volume of 10 *μ*L consisting of 3.75 *μ*L ddH_2_O, 0.25 *μ*L 20x TaqMan Small RNA Assay, 5 *μ*L 2x TaqMan Universal PCR Master Mix, and 1.0 *μ*L RT reaction product. Thermal cycling conditions were enzyme activation and initial denaturation at 95°C for 10 min followed by 40 cycles: 95°C, 15 s; 60°C, 60 s.

For microRNA microarray analysis a total of 15 samples were selected (7 samples with BEN-UTUC, 4 samples with non-BEN-UTUC, and 4 normal kidney samples). Microarray analysis was performed following the Agilent protocol “miRNA Microarray System with miRNA Complete Labeling and Hyb Kit,” version 2.4. We used Agilent SurePrint G3 Human v16 miRNA Array Kit, 8 × 60K. This kit is based on miRBase, release 16.0, and it has 1205 human and 144 human viral targeted probes (Agilent Technologies, Santa Clara, CA, USA) [41]. Agilent's miRNA Microarray System uses cyanine 3-labeled targets to measure miRNA in experimental and control samples. We used a sample input of 160 ng of total RNA. Prior to the dephosphorylation all RNA samples were diluted up to 80 ng/*μ*L in DNase/RNase-free water. Two *μ*L of the diluted RNA was put on ice and CIP master mix (2.0 *μ*L) was added to each sample [CIP master mix consisted of 10x calf intestinal phosphatase buffer (0.4 *μ*L), calf intestinal phosphatase (0.5 *μ*L), and nuclease-free water (1.1 *μ*L)]. The samples were dephosphorylated by incubating the reaction at 37°C in a circulating water bath or heat block for 30 minutes. The samples were denatured by adding 2.8 *μ*L DMSO (100%) and incubated in the thermocycler at 100°C for 5 to 10 minutes. After denaturation, ligation procedure was performed at 16°C in the heat block for 2 hours. For ligation step, 4.5 *μ*L of ligation master mix was added to each sample. Ligation master mix consisted of 10x T4 RNA ligase buffer (1.0 *μ*L), cyanine 3-pCp (3.0 *μ*L), and T4 RNA ligase (0.5 *μ*L). After ligation all samples were purified using microBioSpin chromatography columns-6 (Biorad, Hercules, California, USA). RNase-free water was added to each labeled sample up to a total volume of 50 *μ*L. Then samples were pipetted onto the gel bed and centrifuged at 1000 ×g for 4 minutes. After purification the sample was translucent and slightly pink while volume was close to 50 *μ*L. The samples were then dried and after that they were dissolved in 18 *μ*L nuclease-free water. In the next step, 4.5 *μ*L of 10x GE blocking reagent and 22.5 *μ*L of 2x HiRPM hybridization buffer were added to each sample and incubated at 100°C for 5 minutes. Finally, each sample was dispensed to the appropriate position on the gasket slide prior to hybridization. The assembled slide chamber was placed in rotisserie in a hybridization oven set to 55°C and hybridization rotator at 20 rpm. Hybridization was performed during 20 hours, and afterwards the slides were washed (5 minutes in wash buffer 1 and 5 minutes in wash buffer 2) and dried according to the manufacturer protocol. Scanning of the slides was performed with Agilent Microarray Scanner and data were extracted using Feature Extraction software v.10.5. Statistical analysis of the microRNA expression data was performed using R Bioconductor software and GeneSpring v12.5 software following the user manual. R Bioconductor analysis included moderated *t*-test for differential expression of the miRNAs with log fold change >2 and *P* value < 0.05, while GeneSpring statistics included one-way ANOVA, followed by moderated *t*-test, also set at *P* value < 0.05, and log fold change >2.

Six differentially expressed microRNAs were investigated by qPCR (hsa-miR-205-5p, hsa-miR-210, hsa-miR-224-3p, hsa-miR-373-5p, hsa-miR-99b-3p, and hsa-miR-30a-5p). RNU 6b and RNU 48 miRNAs endogenous controls were used as controls. The methodology included stem-loop RT followed by TaqMan PCR analysis, as described in the upper section. Statistical analyses included *t*-test performed using DataAssist Software v. 3.01.

To explore the functional impact of differentially expressed microRNAs we used DIANA-miRPath v. 3.0 with a focus on pathway union with *P* < 0.05 and microT-CDS threshold set at 0.8. Fisher's exact test (hypergeometric distribution) was used as enrichment analysis (http://snf-515788.vm.okeanos.grnet.gr/dianauniverse/index.php?r=mirpath) [42].

## 3. Results

**Figure 1:**
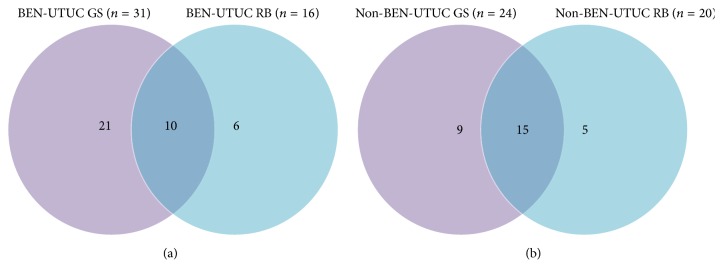
Venn diagrams of differentially expressed microRNAs in BEN-UTUC samples (a) and non-BEN-UTUC samples (b), both in comparison to normal kidney samples and determined by GeneSpring (GS) and R Bioconductor (RB) statistical programs.

Preliminary qPCR analysis with RNU 44 control microRNA revealed that 15 RNA samples were with satisfactory quality for microRNA microarray analysis, including 7 samples with BEN-UTUC, 4 non-BEN-UTUC samples, and 4 nontumor samples. Statistical analysis using R Bioconductor and GeneSpring v12.5 software revealed a number of differentially expressed miRNAs (*P* < 0.05) and at least two log fold changes when BEN-UTUC and non-BEN-UTUC were compared to the normal kidney samples (Figure 1). The analysis using GeneSpring software showed a total of 31 differentially expressed microRNAs, while the R Bioconductor software showed 16 differentially expressed microRNAs in BEN-UTUC samples. Ten microRNAs were differentially expressed with both statistical programs (Figure 1(a)). The comparison between non-BEN-UTUC and normal kidney tissues showed 24 and 20 differentially expressed microRNAs with GeneSpring and R Bioconductor, respectively, of which 15 were detected with both types of software (Figure 1(b)).

Differentially expressed microRNAs (determined both with GeneSpring software and R Bioconductor software) in BEN-UTUC and non-BEN-UTUC tissues are listed in Table 2. Only one microRNA (hsa-miR-205-5p) was differentially expressed among both BEN-UTUC and non-BEN-UTUC samples. Most of the microRNAs were upregulated (7 of 10 in BEN-UTUC and 14 of 15 in non-BEN-UTUC). Only three microRNAs (hsa-miR-127-3p, hsa-miR-154-5p, and hsa-miR-30a-5p) were downregulated among BEN-UTUC, and one microRNA (hsa-miR-663b) was downregulated among non-BEN-UTUC samples.

**Figure 2:**
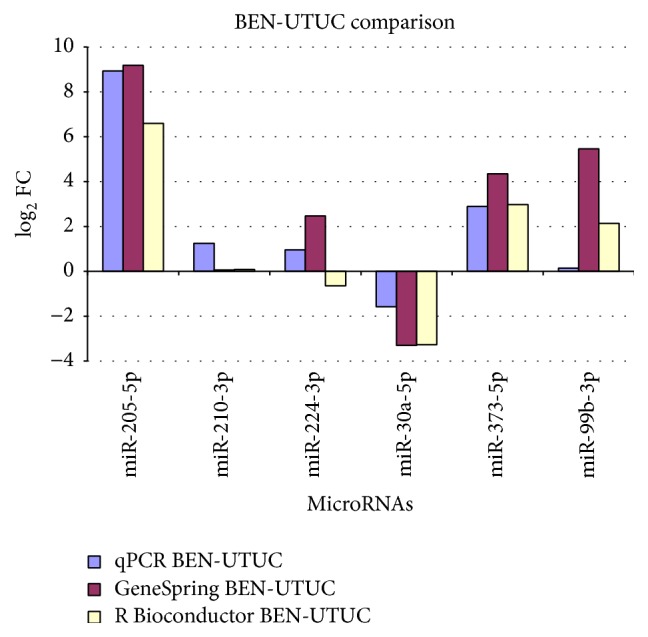
Comparison of qPCR and microarray data in BEN-UTUC samples.

**Figure 3:**
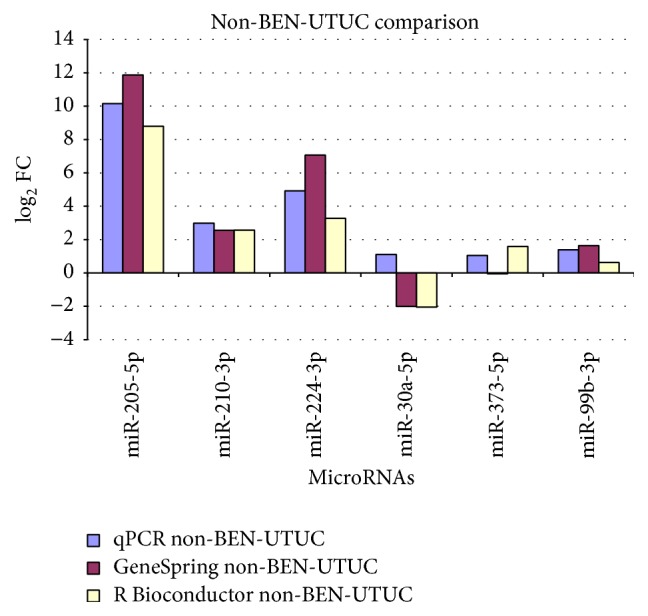
Comparison of qPCR and microarray data in non-BEN-UTUC samples.

With the exception of hsa-miR-99b-3p, the qPCR were in agreement with the microarray results (Figures 2 and 3). In particular, hsa-miR-205-5p was upregulated in both BEN-UTUC and non-BEN-UTUC, and hsa-miR-210-3p and hsa-miR-224-3p were overexpressed with a log FC >2 in non-BEN-UTUC, while hsa-miR-373-5p was overexpressed and hsa-miR-30a-5p was underexpressed in BEN-UTUC samples.

**Figure 4:**
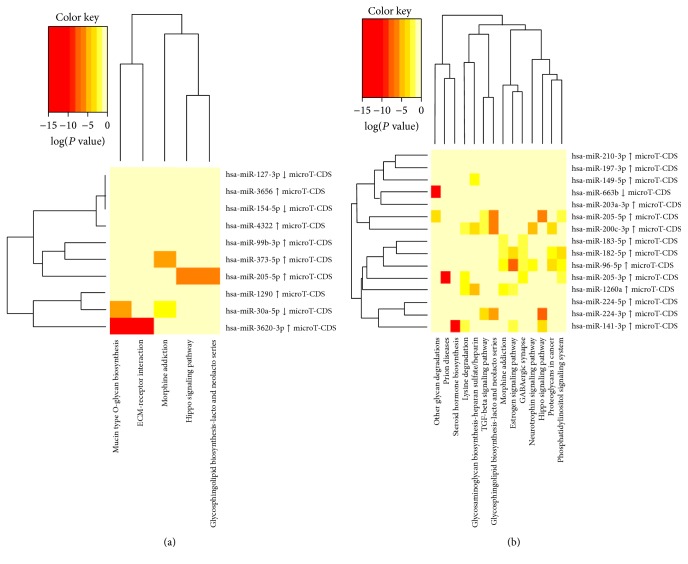
Heat maps of differentially expressed miRNAs and target gene related pathways derived from DIANA-miRPath v.3.0 analysis determined by “pathway union” option in (a) BEN-UTUC and (b) non-BEN-UTUC patients.

In silico analysis of the microRNAs differentially expressed in BEN-UTUC patients (hsa-miR-205-5p, hsa-miR-4322, hsa-miR-99b-3p, hsa-miR-3620-3p, hsa-miR-373-5p, hsa-miR-3656, hsa-miR-1290, hsa-miR-30a-5p, hsa-miR-127-3p, and hsa-miR-154-5p) with DIANA-miRPath version 3.0 (http://snf-515788.vm.okeanos.grnet.gr/dianauniverse/index.php?r=mirpath) [42], using miRNA target predicted in DIANA-microT-CDS v.5.0, revealed several potential pathways. Twenty-nine pathways were revealed using “gene union” option, but only five potential pathways were identified using “pathway union” option (with *P* < 0.05 and microT-CDS threshold at 0.8), in which some of the BEN-UTUC differentially expressed microRNAs are involved. The five pathways identified by “pathway union” option, as well as the microRNAs involved and the targeted genes, are given in Table 3. Heat maps of differentially expressed miRNAs and target gene related pathways for BEN-UTUC and non-BEN-UTUC patients, identified using DIANA-microT-CDS v.5.0 and “pathway union” option, are presented in Figure 4.

## 4. Discussion

This is the first study of microRNA profiling of UTUC associated with BEN. Our initial results showed that different microRNAs are differentially expressed in patients with BEN-UTUC and non-BEN-UTUC. Only one microRNA (hsa-miR-205-5p) was differentially expressed (upregulated) in both groups. Hsa-miR-205-5p is characteristic for different cancer tissues having a diverse role in tumor initiation, progression, and metastasis acting as an oncogene or tumor suppressor [43–49].

In patients with BEN-UTUC we determined three downregulated miRNAs, that is, hsa-miR-127-3p, hsa-miR-154-5p, and hsa-miR-30a-5p. Some studies revealed that hsa-miR-154-5p and hsa-miR-127-3p belong to the cluster of miRNAs located on the 14q32 and they are significantly downregulated in cancers [50–52]. Hsa-miR-30a-5p was found to be downregulated in lung [53] and colorectal [54] and upregulated in ovarian cancer [55]. In our study, seven microRNAs (hsa-miR-1290, hsa-miR-205-5p, hsa-miR-3620-3p, hsa-miR-3656, hsa-miR-373-5p, hsa-miR-4322, and hsa-miR-99b-3p) were upregulated in BEN-UTUC.

Recently a few studies that investigated microRNA profile in nephropathies caused by aristolochic acid in humans and rats have been published [56–58]. Tao et al. [57] compared the expression of microRNA in aristolochic acid nephropathy-UTUC tissues (AAN-UTUC) and non-AAN-UTUC tissues in order to identify the unique gene alterations for AAN-UTUC. They have found 29 differentially expressed microRNAs; the eight most significantly expressed microRNAs are hsa-miR-4795-5p ↓, hsa-miR-488 ↑, hsa-miR-4784 ↓, hsa-miR-330 ↓, hsa-miR-3916 ↓, hsa-miR-4274 ↑, hsa-miR-181c ↓, and hsa-miR-4434 ↑.

Other studies of the carcinogenetic effect of aristolochic acid were performed by Meng et al. [56] and Wang et al. [58]. The study by Meng et al. [56] focuses on determination of microRNAs that could be used as tissue-specific biomarkers for mutagenicity and carcinogenicity produced by aristolochic acid in rats. They found 19 differentially expressed microRNAs (8 upregulated and 11 downregulated) in the kidney, after oral supplementation with aristolochic acid (10 mg/kg body, five times a week for 12 weeks). Among the most significantly differentially expressed upregulated miRNAs they found rno-miR-21-5p and rno-miR-34a-5p, selecting them as potential biomarkers for carcinogenicity and genotoxicity of aristolochic acid, respectively [56].

Wang et al. [58] worked on rats as animal models in order to build a microRNA-gene regulatory network to investigate the molecular dynamics induced by aristolochic acid from a systematic perspective. They analyzed the expression data before and after treatment with aristolochic acid to determine the differentially expressed miRNA and obtained 49 significantly differentially expressed miRNAs with fold change threshold of 1.5 (32 upregulated and 17 downregulated). The most significantly differentially expressed miRNAs were found to be the members of miR-34 family. Others involved miR-21, miR-224, miR-375, and miR-383 [58].

The differentially expressed microRNAs found in our study differ from those presented in these three studies investigating the aristolochic acid nephropathy. Two studies investigated the toxicity of aristolochic acid in rat models [56, 58], making direct comparison not quite suitable. The small number of patients analyzed both in our study and in the study by Tao et al. might be responsible for the difference. Furthermore, we have analyzed the differentially expressed microRNAs in BEN-UTUC and non-BEN-UTUC, both versus normal tissue in order to define microRNAs that could be a signature for BEN-UTUC patients, while Tao et al. [57] investigated expression of microRNA in AAN-UTUC versus non-AAN-UTUC tissues. Also, the flow of disease and the duration of its development differ between BEN and AAN (Chinese herb nephropathy), which occurs more rapidly. Because of this, different pathways and mechanisms might be involved that at the end have the same terminus.

We found 15 differentially expressed microRNAs among non-BEN-UTUC samples, of which only hsa-miR-663b was downregulated and 14 were upregulated (hsa-miR-1260a, hsa-miR-141-3p, hsa-miR-149-5p, hsa-miR-182-5p, hsa-miR-183-5p, hsa-miR-197-3p, hsa-miR-200c-3p, hsa-miR-203a-3p, hsa-miR-205-5p, hsa-miR-205-3p, hsa-miR-210-3p, hsa-miR-224-5p, hsa-miR-224-3p, and hsa-miR-96-5p). Our results are in correlation with the findings by Wei et al. [59] who investigated the possibility of using miRNAs as noninvasive markers in the screening or follow-up of urothelial cell carcinoma (UCC) from upper urinary tract and bladder. They found several microRNAs (miRNA-96, miRNA-182, miRNA-183, miRNA-141, miRNA-30b, miRNA-21, and miRNA-200c) that were overexpressed in high grade UCC.

Pathway analysis revealed five predicted pathways for BEN-UTUC and 14 for non-BEN-UTUC, of which three were mutual for both groups, that is, glycosphingolipid biosynthesis-lacto and neolacto series, Hippo signaling, and morphine addiction pathway (Figure 4). BEN-UTUC specific pathways were mucin type O-glycan biosynthesis and ECM-receptor interaction pathways.

Cellular glycosylation mechanisms and their biosynthetic pathways are very complex and have been shown as fundamental for the changes in glycan processing and divergence. Furthermore, numerous studies have shown that alterations in surface glycans play pivotal roles in cancer initiation and progression [60, 61].

The different members of the glycotransferase (GALNACTs) family were shown to be differentially expressed in malignant tumors compared to normal tissue. The deregulation in the expression of the different GALNACTs allows them to play diverse roles in cancerogenesis. Thus,* GALNT1* is highly expressed in bladder cancer tissues, making it a potential prognostic marker in bladder cancer [62];* GALNT2* is overexpressed in oral squamous cell carcinoma [63] and was also found to play a role in modifying EGFR glycosylation in hepatocellular carcinoma, which contributes to its malignant phenotype [64].* GALNT3* is overexpressed in pancreatic cancer, and its suppression significantly correlates with pancreatic cancer cell growth inhibition [65].* GALNT3* was found to be strongly overexpressed in high grade serous epithelial ovarian cancer, as compared to normal ovarian tissue [66]. In our analysis microRNA (hsa-miR-30a-5p) that is found to be associated with these genes involved in mucin type O-glycan biosynthesis is downregulated implicating their overexpression. The second microRNA involved in this pathway was hsa-miR-3620-3p. This microRNA is upregulated in our analysis suggesting downregulation of the genes that it controls.

ECM-receptor interaction pathway also included the genes controlled by hsa-miR-3620-3p. The study by Li and Zhang [67] represents a probabilistic approach called PanMiRa (Pan-Cancer miRNA-Target Associations) to infer recurrent miRNA-target interactions across 12 cancer types from The Cancer Genome Atlas (TCGA). They found that the targets involved in pan-cancer interactions are enriched for not only cancer pathways such as TGF-b signaling pathways but also pathways related to extracellular matrix (ECM) organization, focal adhesion, and ECM-receptor interactions, all of which are essential pathways in oncogenesis. ECM-receptor interaction pathway belongs to a wider pathway network: phosphatidylinositol 3′-kinase- (PI3K-) Akt signaling pathway that is activated by many types of cellular stimuli or toxic insults and regulates fundamental cellular functions such as transcription, translation, proliferation, growth, and survival [68].

Further studies on larger number of samples from BEN-UTUC are warranted to determine whether these two predicted pathways contribute to the process of BEN and related carcinogenesis.

## 5. Conclusions

To the best of our knowledge, here we present the first microRNA profiling in patients with UTUC from patients living in Balkan endemic nephropathy regions. It also provides further information of the microRNA expression in patients with UTUC from nonendemic regions. An interesting finding is the difference of microRNAs expression among UTUC in patients from BEN and nonendemic regions. This might impose that different mechanisms/pathways could be responsible for the process of carcinogenesis in UTUC in patients from BEN regions.

## Figures and Tables

**Table 1:** Clinical and histopathological data of patients with upper tract urothelial cancer from Balkan endemic nephropathy regions (BEN-UTUC) and nonendemic regions (non-BEN-UTUC).

Sample	Gender	Age	Place of living	Tumor localization	Side	Growth pattern	Grade (low/high)	Stage (low/high)
BEN-UTUC 1	M	79	Endemic region (village Mezgraja)	Renal pelvis	Right	Solid	High	Low
BEN-UTUC 2	M	63	Endemic region (village Tesica)	Ureter	Left	Solid	Low	High
BEN-UTUC 3	M	65	Endemic region (village Mezgraja)	Ureter	Right	Papillary	Low	Low
BEN-UTUC 4	M	71	Endemic region (village Donja Trnava)	Renal pelvis	Right	Papillary	High	Low
BEN-UTUC 5	M	59	Endemic region (village Moravski Bujmir)	Renal pelvis and ureter multifocal	Left	Papillary	Low	Low
BEN-UTUC 6	F	64	Endemic region	Renal pelvis	Left			
BEN-UTUC 7	F	61	Endemic region (village Kocane, Doljevac)	Renal pelvis	Left	Papillary	Low	High
Non-BEN-UTUC 1	F	66	Nonendemic region (city of Nis)	Renal pelvis	Left	Solid	High	High
Non-BEN-UTUC 2	M	39	Nonendemic region (village Balicevac, Merosina)	Renal pelvis and ureter	Right	Papillary	Low	Low
Non-BEN-UTUC 3	F	65	Nonendemic region (city of Nis)	Ureter	Left	Papillary and solid	High	Low
Non-BEN-UTUC 4	M	68	Nonendemic region (city of Nis)	Renal pelvis	Right	Papillary and solid	High	Low
Non-BEN-UTUC 5	F	76	Nonendemic region(village Vukasinovac, Aleksinac)	Renal pelvis	Left	Solid	High	High

**Table 2:** Differentially expressed microRNAs (determined with both GeneSpring software and R Bioconductor software) in BEN-UTUC and non-BEN-UTUC tissues when compared to normal kidney tissues.

*BEN-UTUC*	Log FC (GS)	*P* (GS)	Log FC (RB)	*P* (RB)	*Non-BEN-UTUC*	Log FC (GS)	*P* (GS)	Log FC (RB)	*P* (RB)
*hsa-miR-205-5p * ***↑***	9.184	8.40*E* − 04	6.598	0	*hsa-miR-205-5p * ***↑***	11.872	0.00135	8.798	0
hsa-miR-4322 ↑	5.808	0.006158	2.202	0.00076	hsa-miR-205-3p ↑	9.674	6.72*E* − 06	6.618	0
hsa-miR-99b-3p ↑	5.459	0.003146	2.144	0.00289	hsa-miR-224-5p ↑	7.268	0.015998	4.509	0.00474
hsa-miR-3620-3p ↑	5.073	0.008373	2.438	0.00195	hsa-miR-224-3p ↑	7.068	0.001425	3.278	0.02833
hsa-miR-373-5p ↑	4.349	0.008005	2.974	0.00092	hsa-miR-197-3p ↑	6.800	0.005539	2.211	0.01435
hsa-miR-3656 ↑	2.313	0.006171	2.294	0.00236	hsa-miR-182-5p ↑	6.717	0.002527	4.366	0.00474
hsa-miR-1290 ↑	2.168	0.006006	2.124	0.00374	hsa-miR-183-5p ↑	6.533	0.007597	4.422	0.00699
hsa-miR-30a-5p ↓	−3.299	0.043734	−3.265	0.02983	hsa-miR-96-5p ↑	6.422	0.031363	3.651	0.01527
hsa-miR-127-3p ↓	−4.790	0.036211	−2.104	0.0197	hsa-miR-203a-3p ↑	6.368	0.023474	4.518	0.00969
hsa-miR-154-5p ↓	−4.838	0.008468	−2.027	0.02172	hsa-miR-149-5p ↑	5.905	0.016274	3.306	0.01082
					hsa-miR-141-3p ↑	3.792	0.00317	3.833	0.00207
					hsa-miR-200c-3p ↑	3.655	0.00114	3.678	0.00028
					hsa-miR-1260a ↑	3.306	0.035875	3.349	0.01684
					hsa-miR-210-3p ↑	2.553	0.004245	2.566	0.02018
					hsa-miR-663b ↓	−5.745	0.022896	−2.605	0.04726

↑: upregulated; ↓: downregulated; GS: GeneSpring; RB: R Bioconductor; FC: fold change; *P*: *P* value.

**Table 3:** Pathway analysis of BEN-UTUC differentially expressed microRNAs based on miRNA targets predicted in DIANA-microT-CDS v.5.0.

KEGG^*∗*^ pathway	*P* value	Number of genes	Genes	Number of miRNAs	miRNAs
Mucin type O-glycan biosynthesis (hsa00512)	1.132427*e* − 14	8	GALNT7, GALNT8, GCNT3, GALNT1, GALNT3, GALNT10, GALNT2, GCNT1	2	hsa-miR-3620-3p hsa-miR-30a-5p

ECM^*∗∗*^-receptor interaction (hsa04512)	7.031241*e* − 10	5	THBS1, COL24A1, COL27A1, CD47, COL4A1	1	hsa-miR-3620-3p

Morphine addiction (hsa05032)	0.008499588	17	PRKCA, GNG12, DRD1, PDE4D, PDE1A, GNG10, GABRA5, GNG2, KCNJ6, GABRB1, GNAI2, PDE7A, ADCY9, GNB4, KCNJ3, GABRA2, PRKACB	2	hsa-miR-373-5p hsa-miR-30a-5p

Hippo signaling pathway (hsa04390)	0.01475759	16	YAP1, BMPR1B, FZD6, WWC1, AMOT, PPP2R2D, TP53BP2, FZD3, CDH1, SMAD4, AXIN2, NKD1, RASSF6, DLG2, SMAD1, INADL	1	hsa-miR-205-5p

Glycosphingolipid biosynthesis-lacto and neolacto series (hsa00601)	0.02886044	3	FUT1, ST3GAL6, FUT9	1	hsa-miR-205-5p

^*∗*^KEGG: Kyoto Encyclopedia of Genes and Genomes; ^*∗∗*^ECM: extracellular matrix.
